# Intervention Effect of Electroacupuncture Combined with EPCs Transplantation on the Mice in Aging Model

**DOI:** 10.1155/2015/972749

**Published:** 2015-08-25

**Authors:** Mei Wen

**Affiliations:** ^1^Institute of Acupuncture and Moxibustion, Chinese Academy of Chinese Medical Sciences, Beijing 100700, China; ^2^Affiliated Hospital of Inner Mongolia University for Nationalities, Tongliao 028000, China

## Abstract

The results of this experiment suggested that electroacupuncture promotes the endothelialization of liver endothelial progenitor cells (EPCs) for mice in D-gal model and improves the repair of vascular endothelial function, as well as increasing the vascular endothelial growth factor (VEGF) expression in liver tissue fluorescence and KL protein levels. Also, it reduces the malondialdehyde (MDA) activity and delays vascular aging and even overall aging. Results showed that the in vivo fluorescence intensity for D-gal EA group was significantly lower than that of D-gal group, *P* < 0.05; VEGF fluorescence expression in liver tissue for D-gal EA group was significantly higher than that for D-gal group, *P* < 0.05; KL protein content in liver tissue for D-gal EA group was significantly higher than that for D-gal group, *P* < 0.05; MDA activity for D-gal EA group was significantly lower than that for D-gal group, *P* < 0.05.

## 1. Introduction

With the increase in aging population, the antiaging has become one of the key research topics in the medical field, and it is the basic requirement for enhancing life efficiency and adjusting the dysfunction of vital organs, as well as rebuilding homeostasis. The process of human aging is closely related to Epithelial Cells (ECs) dysfunction and aging is one of the risk factors for atherosclerosis. The apoptosis and senescence of vascular ECs are the first inducement of atherosclerosis, and its occurrence is concerned with the low regeneration capacity of vascular endothelial tissue and ECs aging, as well as the update and apoptosis of vascular ECs [1]. As one part of the overall aging, vascular aging has an important impact on the overall aging. This study aims to slow the development of overall aging through the intervention of vascular aging.

## 2. Materials and Methods

### 2.1. Animals and Grouping

Thirty healthy Kunming male mice of three months of age with body mass of (20 ± 2) g were chosen and randomly divided into three groups of control group, D-gal group, and D-gal EA group, and each group has ten mice. They were provided by Beijing Vital River Laboratory Animal Center. The National Medical Ethics Committee approved all experimental procedures, and the breeding and use of laboratory animals were conducted in accordance with internationally recognized principles.

### 2.2. Aging Animal Model Preparation

After the adaptive feeding of the mice for 7 days, all other groups except the normal one were conventionally disinfected and the model group were treated with nuchal subcutaneous injection of 5% D-gal (0.9% sodium chloride injection dubbed 120 mg/mL concentration of the solution) of 0.125 g/kg/d in a total of 42 consecutive days. The injection was conducted during 9:00 AM to 12:00 PM each day, making the aging model.

### 2.3. EPCs Transplantation

Human embryonic stem cell can develop into an integrated human body, so it belongs to the totipotent stem cell. Each person's body has some stem cells accompanying his whole life. However, the older the people are, the less the quantity of stem cells is. To compensate for the shortage of stem cells, some scientists suggest obtaining stem cells from embryos or fetuses as well as other animals. Antiaging stem cells proved by recent studies include bone marrow mesenchymal stem cells, umbilical cord blood stem cells, adipose stem cells, germ stem cells, and embryonic stem cells [2–7].

This study procured the bone marrow of mice and used adherent culture to get EPCs and then labeled PKH26 in vitro. After the model was completed, 30 mice were placed in a self-manufactured simple container, and their tails are disinfected by povidone-iodine. EPCs suspension (1 × 106 cells) labeled by 1 mL PKH26 was sucked by sterile syringe and was injected into mice body through caudal vein, and eventually EPCs transplantation is completed.

### 2.4. Electric Acupuncture

After the 42 days of D-gal injection, the EPCs were transplanted through the tail vain injection; then the electroacupuncture lasted continuously 14 days within two sessions. In particular, 0.18 × 13 mm of acupuncture needle (Hua TuoTM) was used for the acupuncture treatment on the point of “Taichong,” which is located at the dorsal depression between the first and second hind toes, in depth of 3 mm. The acupuncture needle is connected electroacupuncture apparatus (MBT-1 micro pulse synchronization pulse therapeutic instrument, Zhejiang Electronics Co., Chinese). The electroacupuncture parameters for continuous wave, voltage 4–6 V, frequency 1 Hz, and time 20 min, after the emergence of limbs quiver slightly for continuous electroacupuncture. The other groups were not given any intervention.

### 2.5. In Vivo Imaging Detection of the EPCs in the Liver Tissues of Mice

#### 2.5.1. In Vivo Imaging System

The Maestro in vivo imaging system (CRI, Woburn, MA, USA) consists of charged-couple device (CCD) and liquid crystal tunable filter, and the filter is capable to handle light with the wavelength ranging from 500 nm to 950 nm. The scanning stepper and bandwidth are both 10 nm. The filters of excitation and emission for EPCs are tuned to 530 nm and 600 nm, respectively. The exposure lasts for 10 s. The spectral separation and data collection are performed using Maestro multispectral analysis software. MBT-1 microcomputer pulse synchronization pulse therapy instrumental is used for electronic acupuncture.

#### 2.5.2. In Vivo Video Imaging Method

Mice anesthesia is conducted by injecting 300 *μ*L (2.5 mg/kg) of 2% pentobarbital sodium into mice for each group. After mice anesthesia, EPCs are injected into the tail veins of mice. After that, the mice are placed in the imaging chamber to capture the fluorescence image of EPCs in vivo.

#### 2.5.3. Quantification of Fluorescence Emission Intensity of EPCs in the Livers of Mice

Carestream MISEV5.3.5 software is used to analyze the fluorescence emission intensity of EPCs in the livers of mice from three florescence images. The area and intensity of fluorescence and the area of mice liver are quantified and recorded.

### 2.6. Detection of VEGF Fluorescence Expression in the Livers of Mice

After the in vivo imaging, 30 mice are cut to death by cervical dislocation and the mice bodies are supinely fixed on the pallet. Beneath the xiphoid, skin incision of 1 cm long is cut along the midline of mice, and then the skin of 1 cm long on both rib edges is cut using a scissor to bluntly separate the subcutaneous tissues. After that, the abdominal muscles are cut along the aforementioned surgical route, and the abdominal cavity is opened with special attention to avoid touching large blood vessels and organs. The surrounding blood is wiped away using cotton balls dipped in saline. Then the right lobe of liver is cut off with the specimen size of 1 cm × 1 cm × 1 cm. The liver samples are placed in 4% paraformaldehyde solution (PH 4.7) for more than 24 hours. These liver specimens should be carefully placed in the 4% paraformaldehyde solution without any overstock. The volume of paraformaldehyde solution is 10 times greater than the volume of the liver specimens so that the liquid can freely flow in the specimen bottle. Then the coronal cut of 3 mm thick is made from liver specimens and embedded in paraffin. Paraffin slicing machine is used to make serial sections with the slice of 3~5 mm thick. Olympus fluorescence microscope manufactured in Japan is selected to quantify the VEGF fluorescence expression in the liver specimens of mice.

### 2.7. Detection of KL Protein Levels in the Livers of Mice

Enzyme-linked immunosorbent assay (ELISA) is used to determine the KL protein levels in the livers of mice. The procedure include (1) cutting the remaining liver tissues after removing the 1 cm × 1 cm × 1 cm liver specimens; (2) mixing the remaining liver tissues with ice-cold PBS buffer to make 10% of liver tissue homogenates; (3) placing the 10% of liver tissue homogenates into the centrifuge tube; (4) measuring the supernatant after centrifugation as the KL protein concentration. Measurement is performed using the microplate reader and KL protein assay kits from Nanjing Jiancheng Institute of Biological Engineering. The optical density (OD) of each tube is determined at the wavelength of 450 nm using the microplate reader.

### 2.8. Measurement of MDA Activity in the Liver Tissues of Mice

MDA assay kits are provided by Nanjing Jiancheng Institute of Biological Engineering, and thiobarbituric acid colorimetry method is used to measure the MDA activity in the livers of mice. 100 *µ*L of 10% of the liver tissue homogenate is prepared by mixing the ice-cold PBS buffer with liver tissues. Various reagents are added according to kit instructions and well mixed, and the vortex shaker is closed with the lid. The vortex shaker is bathed in water at 95°C for 40 minutes, and then the vortex shaker is removed from water to cool in running water. After that, the vortex shaker is centrifugated at 3500 r/min for 10 minutes. The supernatant is collected using a pipette and distilled to zero. The OD value is measured at the wavelength of 532 nm for each tube.

### 2.9. Statistical Analysis

The software SPSS 17.0 is used for the data processing and the data is expressed by the average value ± standard deviation ($\overset{-}{X}\pm S$). The comparison between the groups is made with the *t*-test with *P* < 0.05 as the measurement of statistical difference.

## 3. Results

### 3.1. The General Condition of the Mice in Different Groups

The mice in the normal group were observed to have shiny hair and operate flexibly. The weight increased because of considerable capacity of eating. The mice also had normal cauda equina functions. The model group of mice, however, began to show inability to move stably and was in low spirits after 7 days. After 15 days the mice showed less appetite and thusly a loss in weight. The feces of mice were either dry or unshaped and smelt noxiously and the urine is yellow; the feather was dry, shallow with lack of luster; waxing was clearly observed; their spirits were low.

### 3.2. The Condition of D-Gal Model

Observation with naked eye: the livers of the mice in the normal group were observed to be smooth and soft, reddish-brown; the edges were sharp. In the model group, the livers were seen to be larger in size, the colors were dark, and the edges were blunt. The cut plane felt greasy. Congestion was also observed on the surface. The above symptoms were improved after the treatment of electroacupuncture, as is observed from the third group.

**Figure 1:**
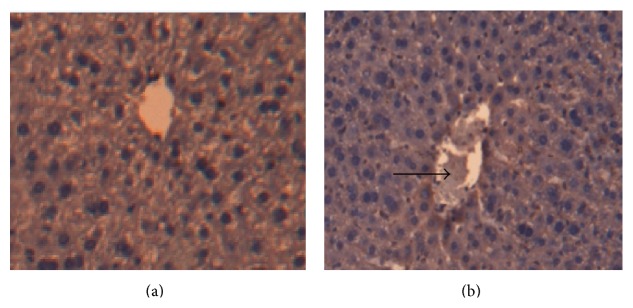
(a) Normal group; (b) model group; radioimmunoassay, ×200; the black arrow points to the inflammatory cells.

Observation by the light microscope: the morphology of the mice liver in the first group was normal. In the radioimmunoassay-staining sample the hepatic cells were radially distributed, centered by the vena centralis. The shapes of the hepatic lobule were clear, and the hepatic cells were in alignment (Figure 1(a)). In the model group, the hepatic cells were swelled and had a disordered arrangement. The mice also suffered from cytoplasm rarefaction, minor steatosis, and edema. Fat droplets with different sizes were seen in the cytoplasm. Part of the steatosis hepatocyte nucleus was extruded by the fat droplets and deformed. Inflammatory cell infiltrations were seen around the central lobule veins (Figure 1(b)).

### 3.3. The Effect on the Mice Liver EPCs

The replicative senescence of cell is one of the most important contents in the aging of human bodies. The replicative senescence and apoptosis of the blood vessel EPCs might be irreversible; however, the acupuncture and moxibustion could help to slow down this process to some extent. The recent researches reveal that aging consists of the interaction of multilevel and Multimolecule; thus it is hard to elaborate with a single doctrine, and the causal relationship could not be divided clearly [8]. Based on the above statements, the development of new antioxidants and the intervening measure to the cell genes such as acupuncture and moxibustion could block the signal passage of oxidative damage of the EPCs, increase the survival rate of the EPCs in the environment of oxidative stress, and repair the endogenous endothelins. This will bring hope to the patients who suffer from ischemic angiopathy.

**Figure 2:**
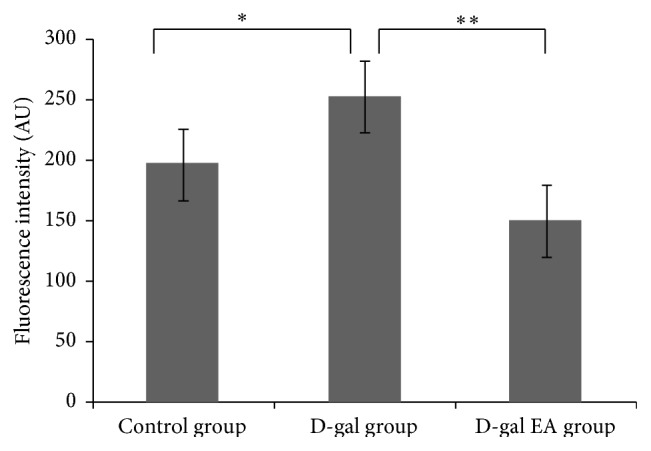
The fluorescence intensity of the livers in different groups after the EPCs treatment with electroacupuncture.

14 days after the EPCs treatment with electroacupuncture with compatibility, the rank of the mean fluorescence intensity of the mouse livers was observed as D-gal group < controlled group < D-gal EA group. Statistical difference (*P* < 0.05) was exhibited between the controlled group and the D-gal group and between D-gal group and D-gal EA group (Figure 2).

**Figure 3:**
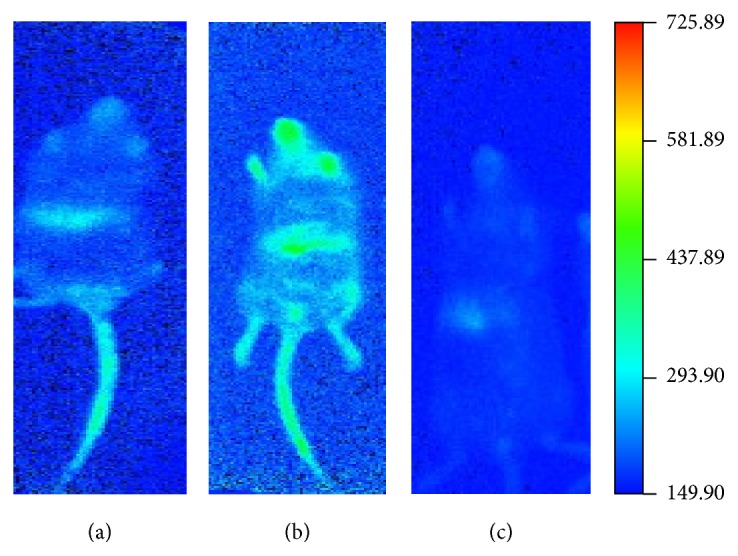
The fluorescence intensity of the livers in different groups after the EPCs treatment: (a) normal group; (b) model group; (c) electroacupuncture group.

After 14-day EPCs treatment of electroacupuncture with compatibility, we observed in the vivo imaging system that the livers of 30 mice exhibited agglomeration of fluorescence, the size of which differed case from case. The fluorescence intensity and the light-emitting area of the livers in the model group were slightly stronger/larger than the normal group, while those of the electroacupuncture group were slightly weaker/smaller than the model group. The general conclusion could be made that the electroacupuncture group exhibited the smallest light-emitting area and weakest fluorescence intensity; in contrast, the model group showed the largest size and the strongest fluorescence (see Figure 3).

### 3.4. The Effect of Electroacupuncture on the Fluorescence Expression of the Liver VEGF

The local vascular damage, ischemia, burn, trauma, hormone, drug, and so on could all force out the EPCs from the marrow. For example, VEGF is the specific assimilation factor and mitogen and chemotactic factor of the vascular endothelial cell. Experiments on both human and animals confirmed the effect of VEGF on the mobilization of the EPCs. VEGF could also increase the targeted home numbers and homing rates, facilitate the swift endothelialization of the damage tissue, and decrease the intimal hyperplasia. It is a new way of auxiliary stem cell transplantation [9, 10].

**Figure 4:**
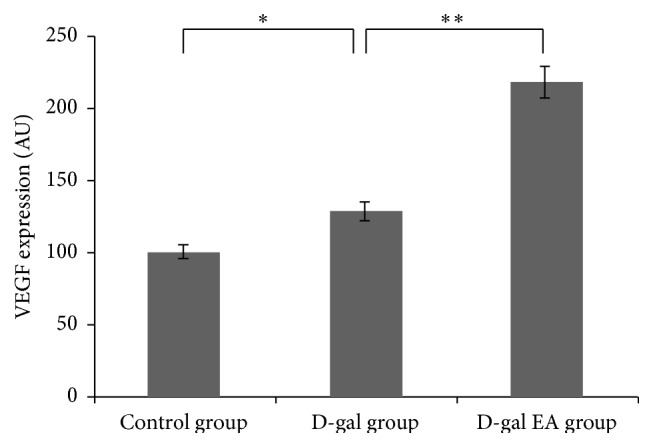
Fluorescence VEGF expression for liver tissues after electrical acupuncture.

**Figure 5:**
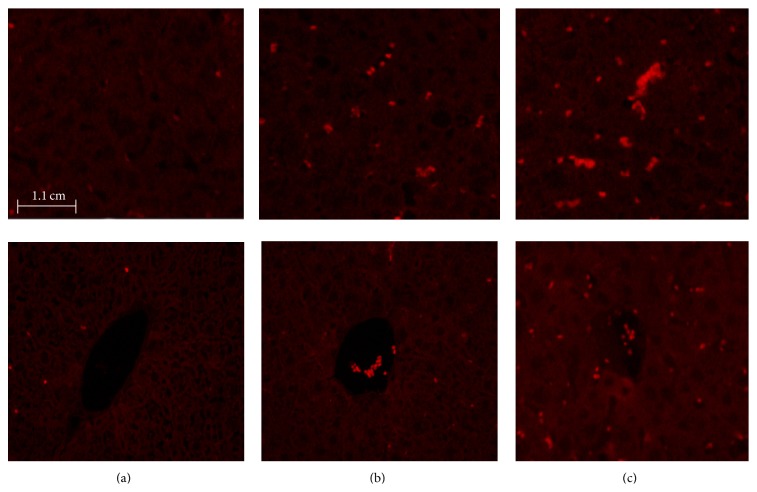
Images of the fluorescence VEGF expression for liver tissues after electrical acupuncture treatment for different groups: (a) control group; (b) D-gal group; (c) D-gal EA group.

The results of this study show that, with the affection of the endogenous factors of ischemia and vascular injury in the D-gal aging mice model and exogenous factor such as electrical acupuncture excitation, EPCs are able to migrate to the site of vascular damage from the tail vein, differentiate into mature endothelial cells, and then secrete VEGF and other cytokines, thereby promoting the angiogenesis, repairing vascular injury, maintaining the environmental balance of the body, and eventually delaying aging (see Figures 4 and 5).

### 3.5. Effects of Electrical Acupuncture on KL Protein Levels in Liver Tissue in Mice

**Figure 6:**
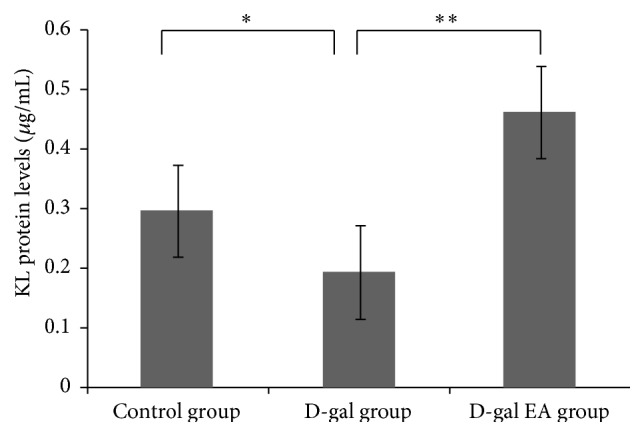
KL protein levels of mice liver tissue after electrical acupuncture.

Xiao et al. tested serum samples from 112 people of different ages from 0 to 91 years old; they found that secreted KL protein decreases as age increases, speculating that KL gene may be a regulator for human aging [11]. Our results suggest that KL protein levels of mice liver tissue significantly decreased in the D-gal group, which is consistent with the results of Xiao. The mice injury in the D-gal group can stimulate EPCs migrating to the site of injury, which increases the number of EPCs KL protein contents, involving in the reendothelialization of the damaged blood vessels (Figure 6).

### 3.6. Effect of Electrical Acupuncture Excitation on the MDA Level in Mice Liver Tissue

**Figure 7:**
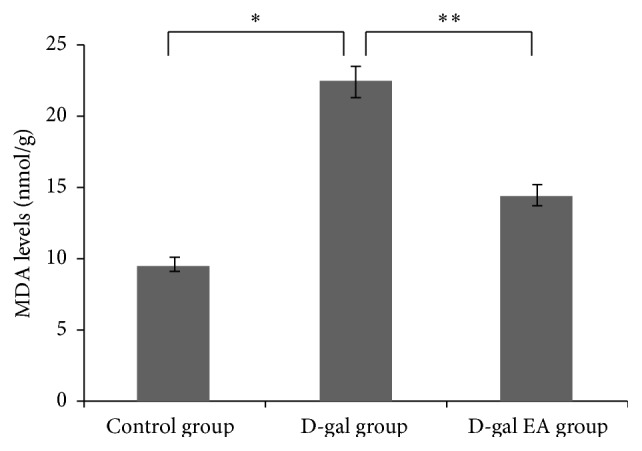
MDA levels in the mice liver tissue after electrical acupuncture.

From the perspective of aging, mitochondrial mutations increase with increasing age, leading to increased accumulation of free radicals, thereby activating the network mechanisms of aging. Under certain circumstances, free radicals can be decomposed to be nontoxic by the enzyme in the body, with no negative effect on humans. However, as the free radicals are extensively generated to exceed the threshold that the enzyme in human body can handle, damaging oxidation will occur in the body. This study uses electrical acupuncture with the combination of EPCs treatment to demonstrate that the MDA level decreased significantly compared to that in the control group, which may facilitate the reendothelialization of vascular damage and improve the balance of the internal environment of the body (Figure 7).

## 4. Discussion

The fundamental reason for the aging of human body as well as the appearance of the wrinkles is the aging process of cells and the decrease in the amount, which is mainly caused by the aging of the stem cells. Like in a production factory, stem cells are the “seed” for the renewal of other tissue cells. Therefore, the aging progresses of the stem cells would seriously weaken the ability of proliferation and differentiation, which leads to the lack of newborn cell supplement and replacement of senescent cells and to dysfunction of the whole body system, making people get older. The stem cell, also known as the cell of origin of universal cells, is a class of self-renewal cells with differentiation potential. In living animals, tissues ensure continued growth and development by means of tissue cell renewal from stem cell division.

The principle of antiaging for stem cell is to activate the body's “self-healing” function through a variety of specific cell injections (including kinds of stem cells and immune cells), which is used to supplement and regulate the diseased cells, to activate required cell functions, to increase the number of normal cells and cell activities, to improve the quality of cells, to prevent and delay diseased cells, and to restore normal physiological function of cells, so as to achieve the recovery and antiaging purposes. Stem cells and progenitor cells preserve the potential of reversibility caused by aging, which might be activated or recovered by appropriate external stimulation [12]. This study attempts to treat the aging mice with D-gal model by the methods of the electroacupuncture against Tsusanli combined with the EPCs injection on caudal vein.

The incidence of cardiovascular disease ranks the first in various diseases associated with aging, which also indicates that vascular aging takes up an important position from another viewpoint [13]. Aging is a progressive decline process of body function after the multicellular organisms grow to maturity. The decrease in the reactivity of stem cells and progenitor cells is an important feature of degeneration induced by aging, mainly showing that the tissue regeneration ability weakens caused by the decline in the proliferation and differentiation capacity of stem cells and progenitor cells with age. Clinical studies have found that the EPCs in the peripheral blood of elderly patients and atherosclerotic patients are largely consumed, leading to the decreased capacity of vascular endothelium regeneration and repair, or the dysfunction of local microenvironment. The results of this study show that the EPCs fluorescence imaging for the living liver of the mice in D-gal aging model intensifies significantly compared with the normal group, which is related with the supplement of EPCs quantity after the EPCs by tail intravenous injection migrate to the injury site [14, 15].

VEGF is the regulatory factor of promoting angiogenesis which has the strongest effect and the highest specificity [16]. As the ECs specific mitogen and chemokine, VEGF can promote ECs proliferation, migration, and vascular endothelium repair, increase vascular permeability, and promote angiogenesis. It can not only promote EPCs mobilization and homing, proliferation, and differentiation, but also directly mobilize bone marrow cells into the peripheral circulation, thus promoting angiogenesis [17, 18]. Angiogenesis does not rely on EPCs solely, but meanwhile, stimulated by the secreted proangiogenic factors, EPCs may be like monocytes and macrophages which can promote arterial generation by secreting cytokines and growth factors [19].

Existing studies have found that the mechanism of EPC promoting angiogenesis and endothelial damage repair mainly has two aspects: first, it can secrete cytokines such as VEGF and promote angiogenesis in ischemic tissue by paracrine; second, it can differentiate into mature endothelial cells by homing, chemotaxis, and gathering in damaged blood vessels. There are also studies finding that some EPC can differentiate into smooth muscle cells and thus participate in the repair of vascular injury [20]. This study shows that acupuncture combined with EPCs therapy promotes the secretion of VEGF, and VEGF in turn regulates EPCs differentiation, migration, and other functions.

KL is a new gene associated with aging, discovered by Kuro-o et al. in 1997 [21]. It is the first aging suppressor gene of overexpression of prolonging life and low expression of accelerated aging in mammal body. The absence of KL gene expression in mice leads to the appearance of various phenotypes similar to human aging, such as arteriosclerosis, osteoporosis, emphysema, life-span shortening, skin and muscle atrophy, cognitive impairment, hearing loss, and movement disorders. This indicates that KL gene has participated in the regulations of diseases related with the body life and aging [11, 22–24]. The extensive biological effects of KL protein decide that it not only plays an important role in antiaging and antioxidative stress mechanisms, but also has close links with the development of many diseases. Recent studies show that KL protein may exist in mammal body as an antiaging hormone [25]. It has broad application prospects in the regulation of calcium and phosphorus metabolism, antiaging, antioxidation, antiapoptosis, protecting organs, and promoting angiogenesis for aged mice and reendothelialization of blood vessel damage [26].

The newly discovered KL protein functions in both membrane-bound and secretory-type ways to achieve its antiaging efficacy. The KL protein is capable of working against the oxidative stress, an imbalance that is associated with the development of a number of diseases. The current research unveils the fact that the mice subjected to the KL genic mutation exhibit many phenotypes that is aging-related, while the overexpression of KL protein is found to improve the symptoms of aging and thus elongate the life span of the mice [27]. Besides, by suppression of the oxidative stress, the KL protein could also reduce the apoptosis of vessel endothelial cells and improve the functioning of the endothelial cell. Thus, the study of KL protein is meaningful to the aging, apoptosis, angiocardiopathy, and metabolic diseases [28, 29]. The current research reveals that electroacupuncture could facilitate the expression of KL protein, which possibly takes part in the process of reendothelialization of the damaged blood vessels.

Theoretically speaking, the reduction of oxidative stress could facilitate the functionality of EPCs, and antioxidative therapy could prevent the EPCs from malfunctioning. The oxidative stress is responsible for the aging and apoptosis of the EPCs and is the initial cause for the loss of EPCs and impaired endothelial function. EPCs are closely related to the oxidative stress. Although EPCs are more resistant to the oxidative than ECs, in the long term existence of oxidative stress, the oxidative stress could mitigate the antioxidase expression. Thus, the oxidative stress is one of the main causes for the change in the number and functionality of EPCs [30].

In 1956, Harman proposed the aging theory of the free radicals, in which the free radicals generated in the process of cellular metabolism are deemed to play an important role in the aging of human body [31]. After certain harmful stimulation, many of the active molecules in the body, such as active oxygen radicals and active nitrogen radicals, are so overoxidized that they exceed the elimination rate of the oxidation. This process destroys the balance of the oxidative system and the antioxidative system, causing damage to all kinds of tissues. The oxidative stress is a by-product of aerobic metabolism [32].

The theory of aging of the free radicals is initially proposed by Harman, an American scientist, in 1956. The process of material metabolism produces the overoxidated free radicals, causing an imbalance inside the body. Of course there exist repair factors inside human body. However, these factors decrease as people get old. When the impairment caused by the free radicals accumulates to a certain level, it could lead to the change in the cell differentiation and even vanishment and at last cause and accelerate aging [33, 34].

Aging is reflected as the oxidation damage of tissues and endocrine disorders. Aged tissues produce large amounts of toxic substances reactive oxygen species, free radicals, and other toxic substances, influencing the expression and activation of cell components. The change of cell components will retard the growth and differentiation of stem cells and progenitor cells and the regeneration and repair of tissues. This study shows that the acupuncture therapy combined with EPCs significantly improves the VEGF expressions, increases the reendothelialization area of injured blood vessels, and retards the process of atherosclerosis. That is because the acupuncture therapy combined with EPCs can promote the mobilization of EPCs in aged mice and the reendothelialization of injured blood vessels and suppress the intimal hyperplasia.

Oxidation stress is a common pathological mechanism for many diseases, and KL protein can protect vessels by inhibiting oxidation stress. The continuous mobilization of EPCs in injured blood vessels after blocking/reducing the production of KL proteins means that KL proteins participate and/or have a great influence on the mobilization of EPCs and that there may be some other unknown mobilization mechanisms of EPCs [35]. In this study, the acupuncture therapy combined with EPCs may prevent vascular injury and even slow down the aging process by increasing the content of KL proteins and inhibiting the activity of MDA.

## 5. Conclusions

Current research shows that aging is the process by which cell activities decrease, finally leading to aging of cells, tissues, and organs. Consequently, aging is the aging process of cells. Aging and regeneration simultaneously exist in organisms, and aging would happen if the aging process is greater than the regeneration process. The aging process of tissues would be delayed and even blocked provided with cells immortalization. Current animal experiments show that various stem cells have the capability of antiaging. Nowadays, cellular senescence is considered as a gradual change process along with the aging of organism body. Differentiated cells in the internal environment often completely lack the capability of redifferentiation and are eventually aged and died due to high degree of differentiations. Fortunately, there are some undifferentiated cells, that is, stem cells, remaining in the bodies throughout the development of animals. The aging of stem cells has a great influence on the aging of human bodies and organisms. As a consequence, the transplantation or injection of human stem cells helps a lot to prevent human aging.

Acupuncture can prevent aging mainly in the following aspects: the neuroendocrine network, blood and circulatory system, and metabolism. There are differences in senescence and diseases between individuals; consequently, the fundamental network mechanisms may be different; for instance, some may be due to the main endocrine recession, and some may be due to dyslipidemia. In this study, acupuncture can promote EPCs endothelial capabilities, inhibit the vessel regenerations of mice according to the D-gal model, and maintain balance in the inner environment after the electroacupuncture treatment combined with EPCs transplantation. All the aforementioned phenomena after the treatment need to be validated by further experiments.
